# Association between *KCNC1* gene polymorphisms and interindividual variability in aerobic capacity following high-intensity interval training

**DOI:** 10.3389/fphys.2026.1825638

**Published:** 2026-07-24

**Authors:** Yan Liu, Jihan Wang, Junren Lai, Wenqi Liu, Duoqi Zhou

**Affiliations:** 1 Department of Sport, Anqing Normal University, Anqing, China; 2 Biology Teaching and Research Group, No. 39 Middle School of Liuzhou, Liuzhou, China

**Keywords:** aerobic capacity, Chinese Han youth, high-intensity interval training, KCNC1, single nucleotide polymorphisms

## Abstract

**Objective:**

To explore the association between single nucleotide polymorphisms (SNPs) of the potassium voltage-gated channel subfamily C member 1 (*KCNC1*) gene and the improvement of aerobic capacity after high-intensity interval training (HIIT), and to screen potential genetic markers sensitive to HIIT.

**Methods:**

A total of 249 Han college students (115 males and 134 females, aged 21.00 ± 0.07 years) were recruited and underwent 12 weeks of HIIT intervention, three times a week. Aerobic capacity indicators were measured before and after the intervention. Genotyping was performed using the Infinium CGA gene chip, and five *KCNC1* gene SNPs were obtained after quality control with PLINK software. Linear regression models were used to analyze the association between SNPs and the training change values (Δ) of each indicator, with gender, age, height and baseline values as covariates.

**Results:**

After 12 weeks of HIIT, VO_2_max/kg, VCO_2_, and VE significantly increased (*P* < 0.01). Submaximal heart rate during running economy (RE) assessment decreased significantly (*P* < 0.01), whereas maximal heart rate did not change significantly (*P* = 0.124). Association analysis revealed that three SNPs were significantly associated with training sensitivity: rs12574348 was suggestively associated with the Δ values of VO_2_max/kg (*β* = -0.9319, *P* = 0.048) and submaximal HR (RE, *β* = 3.069, *P* = 0.020), with CC-type subjects showing the greatest increase in VO_2_max/kg and the greatest decrease in submaximal HR (RE); rs757511 was suggestively significantly associated with the Δ values of VCO_2_ (*β* = 0.1076, *P* = 0.014) and maximal HR (*β* = 1.827, *P* = 0.048), with AA-type subjects showing the greatest increase in VCO_2_ and GG-type subjects showing the greatest decrease in HR_max; rs61882396 was suggestively significantly associated with the Δ value of VO_2_/kg (RE, *β* = -0.7673, *P* = 0.020), with AA-type subjects showing the greatest decrease in VO_2_/kg (RE). None of the above SNPs were significantly associated with baseline indicators (*P* > 0.05).

**Conclusion:**

The polymorphisms of *KCNC1* gene rs12574348, rs757511, and rs61882396 are suggestively significantly associated with the improvement of aerobic capacity indicators after HIIT, and the effects are independent of baseline levels, suggesting potential involvement of *KCNC1* in training-related neuromuscular adaptations, although the exact mechanisms remain to be established, thereby influencing individual sensitivity to HIIT. This gene may serve as a potential genetic marker for predicting the training effect of HIIT.

## Introduction

The primary physiological indicators used to assess aerobic capacity include heart rate (HR), carbon dioxide output (VCO_2_), maximal oxygen uptake (VO_2_max), and the rating of perceived exertion (RPE). Aerobic capacity serves as a critical biomarker of cardiopulmonary function and exercise endurance performance (Oorschot et al., 2023). High-intensity interval training (HIIT) has garnered substantial scientific and clinical interest owing to its time-efficient and potent effects on enhancing aerobic capacity and promoting metabolic adaptations (Atakan et al., 2021). For instance, Chin et al. (2020) implemented an 8-week, low-frequency HIIT protocol in 56 overweight or obese young men and observed statistically significant improvements in both VO_2_max and fat-free mass in the intervention group (*P* < 0.01). Similarly, a 6-week HIIT intervention was shown to confer long-term preservation of elevated VO_2_max levels—sustained for up to four years post-intervention—in previously sedentary older men (Herbert et al., 2021). Nevertheless, interindividual variability in baseline aerobic capacity and responsiveness to HIIT is partly attributable to genetic factors, particularly single nucleotide polymorphisms (SNPs). Evidence indicates that polymorphisms in the PPARGC1A gene are associated with interindividual differences in VO_2_max among middle-aged Japanese men (Nishida et al., 2015); furthermore, a genome-wide association study (GWAS) revealed that Russian elite athletes homozygous for the G allele at rs1052373 in the MYBPC3 gene exhibited significantly higher VO_2_max compared with carriers of the AA or AG genotypes (*P* = 0.005) (Al-Khelaifi et al., 2020). In a cohort of 237 Han Chinese college students undergoing a 12-week HIIT program, Lai et al. (2024) identified a significant association between the ADCY3 rs6753096 variant and differential changes in body fat percentage following training (*P* = 0.0475). Likewise, Yang et al. (2023) reported that the GABRB3 rs17116985 SNP was genome-wide significantly associated with VO_2_max trainability in 228 sedentary adults completing 12 weeks of HIIT (*P* < 5 × 10^−8^); additionally, rs474377, rs9365605, and rs17116985 collectively accounted for 11%, 9%, and 6.2% of the interindividual variance in VO_2_max response, respectively.

The potassium voltage-gated channel subfamily C member 1 (*KCNC1*) gene is located at chromosome 11p15.1 and encodes the Kv3.1 voltage-gated potassium channel protein. The Kv3.1/KCNC1 signaling pathway is highly expressed throughout the ascending auditory pathway, fast-spiking interneurons (FSIs) across multiple brain regions, and GABAergic inhibitory interneurons (Spinelli et al., 2024). Functional studies demonstrate that motor neurons expressing Kv3.1 generate high-frequency, temporally precise action potential trains, thereby maintaining narrow calcium influx windows at presynaptic terminals—ensuring reliable acetylcholine (ACh) release, robust endplate potentials, and consistent skeletal muscle fiber contraction (Kaczmarek and Zhang, 2017). Preclinical evidence further supports a modulatory role for GABA in peripheral neuromuscular transmission, with widespread expression detected during early neuro-muscular development in murine models (Sibgatullina and Malomouzh, 2020). Clinically, the KCNC1 p.R320H missense mutation is causally linked to myoclonic epilepsy and progressive ataxia (Oliver et al., 2017). Collectively, these findings suggest that gain-of-function or loss-of-function variants in *KCNC1* gene may alter Kv3.1 biophysical properties and downstream neuromuscular signaling fidelity, ultimately influencing motor performance and exercise-related physiological adaptations.

Despite growing recognition of KCNC1’ s functional relevance to neuromuscular excitability and synaptic precision, empirical evidence linking *KCNC1* genetic polymorphisms to interindividual differences in aerobic capacity responsiveness to HIIT remains scarce. Accordingly, this study investigates the association between common SNPs in the *KCNC1* gene and the magnitude of improvement in aerobic capacity—specifically VO_2_max—among healthy young adults undergoing standardized HIIT. We hypothesize that *KCNC1* variants may serve as predictive genetic markers of HIIT trainability. Findings from this investigation will advance mechanistic understanding of the genetic architecture underlying exercise adaptation and inform the development of genetically informed, personalized exercise prescription strategies.

## Methods

### Subjects inclusion and exclusion criteria

A total of 259 Han Chinese undergraduate and graduate students were recruited via voluntary enrollment for this study, which was conducted between September 2018 and December 2022. Eligible participants were required to meet all of the following inclusion criteria: (1) Han Chinese ethnicity; (2) age between 18 and 30 years; and (3) no prior formal or systematic sports training. Exclusion criteria included: (1) presence of any chronic medical condition—including cardiovascular, metabolic (e.g., diabetes), neurological, musculoskeletal, oncological, or autoimmune disorders; (2) clinically diagnosed hypertension (resting systolic blood pressure ≥140 mm Hg and/or diastolic blood pressure ≥90 mm Hg); (3) any acute or chronic physical or medical condition likely to interfere with experimental procedures or outcome assessments; (4) engagement in high-intensity physical activity exceeding three hours per week of moderate-to-vigorous intensity exercise; (5) current use of tobacco, excessive alcohol consumption (defined as >14 standard drinks/week for men or >7 for women), habitual sleep deprivation (i.e., average nightly sleep <6 hours over the preceding month), or recent (within the past three months) use of opioids, systemic corticosteroids, or psychotropic medications. Prior to participation, all individuals underwent standardized sports risk screening and provided written informed consent. Throughout the study period, participants were instructed to maintain their usual lifestyle routines, refrain from initiating any additional structured exercise programs, and avoid consuming medications or dietary supplements known to influence physiological or cognitive outcomes under investigation. Ultimately, 249 participants satisfied all eligibility requirements and completed the full protocol, including all scheduled training sessions and assessments. Baseline characteristics of subjects are summarized in **Table 1**. The manuscript was performed in accordance with the ethical standards of the Helsinki Declaration and the study protocol and informed consent documents received ethical approval from the Institutional Review Board of Beijing Sport University (Approval No: 2018018H).

**Table 1:** Basic information of the subjects.

Group	N	Age (year)	Height (cm)	Weight (kg)	BMI (kg/m^2^)
Male	115	21.13 ± 0.11	173.56 ± 0.54	67.12 ± 1.10	22.07 ± 0.31
Female	134	20.90 ± 0.09	160.21 ± 0.50	53.05 ± 0.62	20.50 ± 0.21
Total	249	21.00 ± 0.07	166.37 ± 0.56	59.55 ± 0.75	21.22 ± 0.19

### Exercise intervention

#### Quantitative load exercise heart rate test

In this study, a cycle ergometer was utilized to administer standardized submaximal exercise testing. Concurrently, cardiopulmonary and cardiovascular responses were assessed using a Cortex MetaMax 3B gas exchange analyzer (Cortex Biophysik GmbH, Leipzig, Germany) and a Polar H10 heart rate monitor (Polar Electro Oy, Kempele, Finland). The exercise protocol was standardized by sex: male participants performed cycling at a constant workload of 100 W, whereas female participants cycled at 75 W. All participants maintained a fixed pedaling cadence of 60 revolutions per minute (rpm), paced by an auditory metronome. During data analysis, oxygen uptake (VO_2_) and heart rate (HR) values recorded during the final 3 minutes of the steady-state phase—defined as the period of stable physiological response following initial exercise adaptation—were extracted and averaged. Heart rate values exceeding ± SD from the group mean were excluded in accordance with established outlier detection criteria. The resulting parameters included absolute VO_2_ (L·min^−1^), relative VO_2_ (mL·kg^−1^·min^−1^), and HR (bpm), which collectively informed the assessment of running economy (RE).

#### VO2 max test

The test protocol employed an incremental exercise protocol, with baseline workloads established at 50 W for male participants and 40 W for female participants, followed by progressive increments of 25 W and 20 W, respectively, every two minutes. A metronome was utilized throughout the test to standardize cadence at 60 revolutions per minute (rpm). During the test, heart rate was continuously monitored using a validated Polar heart rate monitor, and the Borg Rating of Perceived Exertion (RPE) scale was administered at the conclusion of each workload stage. Post-test, individualized training intensities were prescribed based on the empirically determined VO_2_max value.

### HIIT exercise intervention program

The exercise intervention in this study consisted of a 12-week HIIT program, delivered three times per week. Each session comprised alternating high-intensity intervals (performed at 80–90% of VO_2_max) and active recovery intervals (performed at 50–55% of VO_2_max). A detailed description of the session structure and progression is provided in **Table 2**. To ensure adherence to prescribed intensities, participants wore Polar heart rate monitors throughout all training sessions, and real-time heart rate feedback was employed to maintain physiological effort within the target heart rate zones corresponding to the designated intensity ranges. The HIIT training is conducted on the treadmill. During the high-intensity phase, the speed is set at 80% - 90% of the VO_2_max corresponding running speed, and during the recovery phase, the speed is set at 50% - 55% of the corresponding running speed.

**Table 2:** HIIT training program.

Time	High-intensity running	Interval brisk walking	Number of sets	Training frequency
1week	15s	15s	56 sets	3 times/week
2week	30s	30s	28 sets	3 times/week
3week	1min	1min	14 sets	3 times/week
4week	2min	2min	7 sets	3 times/week
5-12week	4min	3min	4 sets	3 times/week

### Genomic data quality control and genotype imputation

A total of 5 mL venous blood was collected from each participant. Genomic DNA was isolated from peripheral blood leukocytes using the Tiangen Genomic DNA Extraction Kit (Tiangen Biotech Co., Ltd., Beijing, China). Whole-genome genotyping was performed using the Illumina Infinium Global Screening Array (GSA) v2.0 (Illumina, Inc., San Diego, CA, USA), yielding genotype data for all 249 enrolled participants. Standardized quality control (QC) procedures were applied to the autosomal genotype data, following established protocols (Marees et al., 2018). Specifically, SNPs were excluded if they exhibited: (i) a missing genotype rate exceeding 3% (n = 31,912); (ii) a minor allele frequency (MAF) < 1% (n = 1,412); or (iii) significant deviation from Hardy–Weinberg equilibrium (HWE; *P* < 1 × 10^−6^, n = 679). Additionally, samples were excluded based on: (i) overall call rate < 95% (n = 3); and (ii) outlier status identified via both heterozygosity-based outlier detection (samples deviating >3 standard deviations from the mean heterozygosity; n = 5) and principal component analysis (PCA) (samples positioned >3 standard deviations from the mean along the top two principal components; n = 2). After QC, high-quality genotype data from 249 samples across 391,200 autosomal SNPs were retained for downstream analyses.

### Statistical analysis

Aerobic capacity data were assessed for normality using SPSS 26.0 (IBM Corp., Armonk, NY, USA). Paired-samples t-tests were conducted to evaluate within-subject changes in aerobic capacity from baseline to post-intervention. SNP quality control was performed using PLINK v1.09, with inclusion criteria set at minor allele frequency (MAF) > 0.05, genotyping call rate > 90%, and Hardy–Weinberg equilibrium (HWE) p-value > 0.05. Subsequently, association analyses were carried out between eligible SNPs and individual responsiveness to HIIT in terms of aerobic capacity improvement. This study is based on scientific hypotheses and selects the *KCNC1* gene in advance. Candidate gene association study was conducted using PLINK v1.9 under an additive genetic model (ADD), with sex, baseline heart rate, and the top five principal components (PCs) derived from genomic principal component analysis (PCA) included as covariates. Between-group comparisons for normally distributed continuous variables were performed using one-way ANOVA with least significant difference (LSD) *post hoc* tests; statistical significance was defined as *p* < 0.05. Continuous variables are presented as mean ± SD. To account for multiple testing across SNPs, the Bonferroni-corrected significance threshold was calculated as α = 0.05/N, where N denotes the total number of SNPs tested.

## Results

### Informations of *KCNC1* gene SNPs

The final gene expression microarray analysis identified 11 SNPs within the *KCNC1* gene. Following stringent quality control and filtering using PLINK software—based on standard criteria including MAF ≥ 0.05, call rate ≥ 95%, and HWE p-value > 1×10^−6^—five SNPs were retained for downstream association analyses. The genomic coordinates, alleles, MAFs, and functional annotations of these five SNPs are summarized in **Table 3**. To account for potential confounding effects on aerobic capacity phenotypes, multivariable linear regression models were fitted with height, age, sex, and baseline aerobic capacity measures (e.g., VO_2_max/kg, VCO_2_, HR) included as covariates. Under an ADD model, statistically significant associations (*P* < 0.05) were observed between: (i) rs757511 and both VCO_2_ and heart rate (HR); (ii) rs12574348 and VO_2_max/kg as well as HR_ssubmax (RE); and (iii) rs61882396 and VO_2_/k (RE). No significant associations were detected for the remaining two SNPs after multiple-testing correction (*P* > 0.05). Importantly, all three significant associations remained robust following sensitivity analyses that excluded the aforementioned covariates—confirming their independence from baseline anthropometric and physiological variables. Subsequent sensitivity analyses further evaluated the stability of these associations across varying baseline values and in response to training intervention effects.

**Table 3:** Information on *KCNC1* gene SNPs included.

CHR	Loci	SNP	Minor allele	Major allele	MAF	Geno-freq	H-W *P*	dbSNP func- annot
11	17759196	rs60835408	T	C	0.1345	4/59/186	1	intronic
11	17768692	rs10832855	T	C	0.09639	4/40/205	0.2593	intronic
***11***	***17774473***	***rs757511***	***A***	***G***	***0.3112***	***25/105/119***	***0.7692***	***intronic***
***11***	***17795729***	***rs12574348***	***T***	***C***	***0.2048***	***12/78/159***	***0.5593***	***3’-UTR***
***11***	***17800923***	***rs61882396***	***A***	***C***	***0.1888***	***9/76/164***	***1***	***intronic***

Italicized and bolded sites indicate significant differences in the association analysis.

### *KCNC1* gene polymorphism and baseline aerobic capacity

As presented in **Table 4**, no statistically significant differences were observed in baseline aerobic capacity among subjects across the KCNC1 polymorphic loci rs12574348, rs757511, and rs61882396 (*P* > 0.05).

**Table 4:** Correlation between SNPs and baseline aerobic capacity.

Phenotype	SNP	Genotype	N	Baseline value	Effect allele	TEST	*β*	*P*
VO_2_max/kg (ml/min/kg)	rs12574348	CC	159	32.84 ± 0.51	T	ADD	-0.4013	0.4703
		CT	78	32.12 ± 0.70				
		TT	12	30.66 ± 1.47				
VCO_2_ (L/min)	rs757511	GG	119	2.30 ± 0.03	A	ADD	-0.003909	0.9211
		AG	105	2.23 ± 0.03				
		AA	25	2.19 ± 0.13				
HR_max (bmp)	rs757511	GG	119	182.69 ± 6.94	A	ADD	0.2968	0.8409
		AG	105	181.80 ± 0.84				
		AA	25	183.61 ± 1.74				
VO_2_/kg(RE) (ml/min/kg)	rs61882396	CC	164	22.22 ± 0.28	A	ADD	-0.6498	0.0836
		AC	76	21.90 ± 0.42				
		AA	9	20.02 ± 1.69				
HR_submax (RE)	rs12574348	CC	159	142.06 ± 1.57	T	ADD	2.736	0.1262
		CT	78	145.51 ± 1.90				
		TT	12	148.42 ± 2.61				

### The impact of HIIT on aerobic capacity

**Table 5** presents the physiological responses following 12 weeks of HIIT. Paired t-tests showed that after 12 weeks of HIIT, VO_2_max/kg, VCO_2_, and VE were significantly increased (*P* < 0.01). HR_submax (RE) test decreased significantly (*P* < 0.01), whereas HR_max did not change significantly (*P* = 0.124).

**Table 5:** HIIT improves aerobic capacity.

Aerobic capacity	Baseline	Training	*P*
VO_2_max/kg (ml/min/kg)	32.51 ± 0.40	36.83 ± 0.44	0.000**
VCO_2_ (L/min)	2.26 ± 0.04	2.84 ± 0.05	0.000**
HR_max (bmp)	182.41 ± 0.96	180.95 ± 0.63	0.124
VE (L/min)	75.11 ± 1.47	85.35 ± 1.60	0.000**
VO_2_/kg(RE) (ml/min/kg)	22.03 ± 0.23	22.33 ± 0.22	0.186
HR_submax (RE) (bmp)	143.43 ± 1.17	137.70 ± 1.06	0.000**

*P < 0.05; **P < 0.01.

### *KCNC1* gene polymorphism and the effects of HIIT on aerobic capacity

Linear regression analyses were performed using PLINK to assess associations between SNPs and training-induced changes in physiological parameters, with height, age, sex, and baseline values included as covariates. As presented in **Table 6**, the following statistically significant associations were observed: (i) rs12574348 was significantly associated with the training effect on VO_2_max/kg; the CC genotype conferred the greatest improvement, with CC homozygotes exhibiting a significantly larger post-HIIT increase in VO_2_max/kg compared to carriers of the T allele; (ii) rs757511 was significantly associated with the training effect on VCO_2_; the AA genotype was associated with the largest absolute increase, significantly exceeding that observed in GG and AG carriers; (iii) rs757511 was also significantly associated with the training effect on HR; the GG genotype was linked to the greatest reduction in HR following HIIT, significantly greater than the reduction observed in AA carriers; Notably, The apparent increase in HR_max among AG heterozygotes (Δ = +2.55 ± 2.58 bpm) should be interpreted with caution due to large within-group variability and potential outlier influence. Sensitivity analysis excluding individuals with ΔHR_max > 5 bpm attenuated the mean change toward zero; (iv) rs61882396 was significantly associated with the training effect on resting VO_2_/kg (RE); the AA genotype was associated with the largest decrease, significantly exceeding the reductions observed in AC and CC carriers; (v) rs12574348 was further significantly associated with the training effect on resting HR_submax (RE); the CC genotype was associated with the greatest reduction in HR_submax (RE), significantly greater than that observed in CT and TT carriers. To assess the impact of extreme BMI or baseline physical fitness on the stability of genetic associations, we excluded individuals with a BMI less than 18.5 or greater than 28 (n = 0; there were no obese individuals in this sample), as well as those with a baseline VO_2_max/kg lower than or higher than the mean ± 2SD (n = 0). After further excluding 5% of individuals at each end of BMI and VO_2_max/kg (approximately 25 individuals in total), we conducted an additive regression analysis again. The results showed that the effect size and significance level of rs12574348 and ΔVO_2_max/kg (*β* = -0.89, *P* = 0.052), rs757511 and ΔVCO_2_ (*β* = 0.10, *P* = 0.018), and rs61882396 and ΔVO_2_/kg (RE) (*β* = -0.73, *P* = 0.025) were largely consistent with the analysis of the entire sample (**Table 6**). Therefore, the association results of this study are relatively robust for variations in BMI and physical fitness within the normal range.

**Table 6:** *KCNC1* gene polymorphism and aerobic capacity in response to HIIT.

Phenotype	SNP	Genotype	N	ΔChange	Effect allele	TEST	*β*	*P*
VO_2_max/kg (ml/min/kg)	rs12574348	CC	159	4.63 ± 0.40	T	ADD	-0.9319	0.04816*
		CT	78	4.03 ± 0.53				
		TT	12	2.20 ± 0.96				
VCO_2_ (L/min)	rs757511	GG	119	0.56 ± 0.04	A	ADD	0.1076	0.01352*
		AG	105	0.56 ± 0.55				
		AA	25	0.84 ± 0.12				
HR_max (bmp)	rs757511	GG	119	-3.27 ± 1.00	A	ADD	1.827	0.04841*
		AG	105	2.55 ± 2.58				
		AA	25	-2.78 ± 1.47				
VO_2_/kg(RE) (ml/min/kg)	rs61882396	CC	164	0.54 ± 0.31	A	ADD	-0.7673	0.02026*
		AC	76	0.44 ± 0.48				
		AA	9	-0.61 ± 1.36				
HR_submax (RE) (bmp)	rs12574348	CC	159	-6.22 ± 1.37	T	ADD	3.069	0.01964*
		CT	78	-1.63 ± 2.55				
		TT	12	-2.83 ± 1.68				

*P < 0.05; **P < 0.01.

### Analysis results of %Δ in relation to the residualized training response

We separately calculated %Δ and the residualized reaction, and then repeated the additive regression analysis, the results are shown in **Table 7**. Most of the correlations remain consistently in the same direction in %Δ and residualized reactions, with P-values being similar or slightly higher.

**Table 7:** **%**Δ and residualized training response analysis.

SNP	Phenotype	Standardized	β (SE)	*P*
rs12574348	ΔVO_2_max/kg	Δ	-0.93 (0.47)	0.048
		%Δ	-2.84 (1.42)	0.046
		Residual	-0.89 (0.45)	0.050
rs757511	ΔVCO_2_	Δ	0.107 (0.043)	0.0135
		%Δ	4.72 (2.01)	0.019
		Residual	0.102 (0.042)	0.016
rs757511	ΔHR_max	Δ	1.83 (0.92)	0.048
		%Δ	0.96 (0.51)	0.062
		Residual	1.69 (0.91)	0.066
rs61882396	ΔVO_2_/kg(RE)	Δ	-0.767 (0.327)	0.020
		%Δ	-3.48 (1.55)	0.025
		Residual	-0.720 (0.321)	0.026
rs12574348	ΔHR_submax(RE)	Δ	3.069 (1.30)	0.0196
		%Δ	2.10 (0.92)	0.024
		Residual	2.91 (1.27)	0.022

### Non-additive genetic model test

We used PLINK v1.9 to separately fit the dominant model (dominant: AG+AA vs. GG) and the recessive model (recessive: AA vs. AG+GG), and compared them with the additive model (**Table 8**). To investigate the possible non-additive effect between rs757511 and ΔHR_max, we separately fitted the dominant model (AG+AA vs. GG) and the recessive model (AA vs. AG+GG). The dominant model showed that individuals carrying at least one A allele had a greater HR_max variation compared to the GG homozygotes (*β* = 2.945, SE = 1.41, *P* = 0.038), but this effect was mainly driven by the extreme values of the AG heterozygotes. The recessive model was not significant (*β* = -2.456, SE = 1.56, *P* = 0.117). The additive model (*P* = 0.048) and the dominant model’s significance both originated from the abnormal high variability of the AG subgroup, rather than stable genotype effects. Therefore, the current data do not support a clear dominant or recessive genetic pattern.

**Table 8:** Non-additive genetic model prediction results.

Model	Control group	β (SE)	*P*
ADD	A	1.827 (0.92)	0.0484
DOM	AG+AA vs. GG	2.945 (1.41)	0.0382
REC	AA vs. AG+GG	-2.456 (1.56)	0.117

### Statistical power test report

The adjusted significance threshold after Bonferroni correction is:α’ = 0.05/30 ≈ 0.00167. The P-values reported in this article are all much higher than 0.00167 and none of them have reached the strict significance level after Bonferroni correction. The current results have a relatively high risk of false positives and should be regarded as exploratory and hypothesis-generating findings rather than conclusive conclusions.

We conducted a post-hoc power analysis using Quanto v1.2.4, and the results are shown in **Table 9**. At the α = 0.05 (uncorrected) level, for SNPs with MAF ≈ 0.19 and effect size R² = 5%, the power of this study was approximately 68%, which was acceptable but relatively low; for the rs757511 with a higher MAF (0.31), the power was approximately 75%. At the Bonferroni-corrected significance level of α = 0.00167, the power is less than 10%.

**Table 9:** Statistical power test results.

R²	MAF	N	α = 0.05	α = 0.00167
5%	0.19	249	~0.68	<0.10
5%	0.31(rs757511)	249	~0.75	<0.12
3%	0.19	249	~0.45	<0.08

MAF, Select the lowest MAF value of the three positive SNPs, which is approximately 0.1888 (rs61882396); R², The contribution of a single SNP to the VO_2_max training response is approximately 6% to 11%, and a conservative estimate would be 5%.

## Discussion

### Association between *KCNC1* gene polymorphism and baseline aerobic capacity

The explanatory power of individual SNPs for complex polygenic traits is typically modest (Speed et al., 2017; Ge et al., 2019). As presented in **Table 4**, the estimated regression coefficient (*β*) for rs12574348 is −0.4013 (*P* = 0.4703), and for rs61882396 it is −0.6498 (*P* = 0.0836). Although the negative effect estimates suggest a potential trend toward reduced aerobic capacity among carriers of the T allele (rs12574348) or A allele (rs61882396), neither association attained statistical significance at the conventional α = 0.05 threshold. These findings are consistent with the hypothesis that these variants represent minor-effect loci, whose detection requires substantially increased statistical power—typically achieved through larger sample sizes (Shi et al., 2018; Okamura et al., 2021). Notably, while none of the three SNPs examined herein demonstrated a statistically significant association with baseline VO_2_max/kg, their consistent directional effects—e.g., a stepwise decline in mean VO_2_max/kg across rs12574348 genotypes (CC > CT > TT)—raise the possibility that these loci modulate responsiveness to endurance training rather than constituting determinants of resting physiological capacity. This interpretation aligns closely with the established molecular function of the Kv3.1 potassium channel, encoded by *KCNC1*, which regulates neuronal and muscular excitability dynamics in activity-dependent contexts. Furthermore, prior evidence regarding *KCNC1* polymorphisms and endurance-related phenotypes reveals population-specific patterns: an association with VO_2_max was reported in individuals of African ancestry, but failed to replicate in cohorts of European and Hispanic descent (Muniz-Aragunde, 2005). Collectively, these observations support a model wherein KCNC1’s functional impact on aerobic traits may be contingent upon genetic background or physiological state—rendering the nonsignificant baseline associations observed in the present study biologically plausible and methodologically expected.

### The impact of HIIT on aerobic capacity

The statistically significant improvement in VO_2_max/kg (*P* < 0.01) constitutes a primary physiological indicator of HIIT efficacy. At the molecular and systemic levels, this enhancement arises from coordinated adaptations in skeletal muscle oxidative metabolism and cardiac output capacity:

(i) Intermittent high-intensity exercise potently activates the AMP-activated protein kinase (AMPK)–peroxisome proliferator-activated receptor gamma coactivator 1α (PGC-1α) signaling axis in skeletal myocytes. As the central regulator of mitochondrial biogenesis, PGC-1α upregulates nuclear respiratory factors (NRF-1 and NRF-2) and mitochondrial transcription factor A (TFAM), thereby stimulating mitochondrial DNA replication and the expression of oxidative phosphorylation complexes (Ito et al., 2024). Consequently, mitochondrial density and intrinsic oxidative capacity per unit muscle mass increase, facilitating greater oxygen extraction and utilization during maximal effort.

(ii) Recurrent hypoxic–reperfusion cycles induced by high-intensity muscular contractions promote local accumulation of hypoxia-inducible factor 1α (HIF-1α) in skeletal muscle, which transcriptionally activates vascular endothelial growth factor (VEGF) expression and stimulates capillary angiogenesis (Lee et al., 2015; Ba et al., 2021; Lei et al., 2024). Concurrently, the significant rise in V̇E (*P* < 0.01) reflects respiratory system adaptation to elevated metabolic demand. Evidence suggests HIIT may modulate chemosensitivity—particularly in central respiratory centers and peripheral carotid bodies—to CO_2_ and H^+^, thereby enhancing ventilatory efficiency and minimizing redundant ventilation (Salazar-Martínez et al., 2018).

In the RE assessment, HR_submax decreased significantly (*P* < 0.05), whereas VO_2_/kg (RE) remained unchanged. RE represents an integrative outcome of metabolic efficiency, cardiopulmonary function, biomechanical optimization, and neuromuscular coordination (Barnes and Kilding, 2015). Although HIIT enhances mitochondrial efficiency, suboptimal running mechanics—including excessive vertical oscillation or insufficient lower-limb stiffness—may counteract metabolic gains and limit improvements in RE (Saunders et al., 2004). Furthermore, the *KCNC1* gene encodes the voltage-gated potassium channel Kv3.1, which governs high-frequency firing fidelity in motor neurons. Emerging evidence indicates that *KCNC1* polymorphisms may influence neural drive recruitment patterns, thereby contributing to interindividual variability in RE (Ozaíta et al., 2002).

### *KCNC1* gene polymorphism and the effects of HIIT on aerobic capacity

(i) Association between rs12574348 and VO_2_max/kg training response: The rs12574348 polymorphism may modulate the biophysical properties of the Kv3.1 potassium channel—specifically, its current density and gating kinetics. The observed association between rs12574348 and the training-induced change in VO_2_max/kg is statistically suggestive but does not directly inform mechanism. Based on the known role of Kv3.1 channels in enabling high-frequency firing in motor neuron (Coletti et al., 2022; Hand et al., 2022), it is hypothesized that the CC genotype might enhance channel activity, thereby supporting more sustained neural drive during HIIT (Martinez-Valdes et al., 2018; Pengam et al., 2023). This could, in theory, promote greater recruitment of high-threshold motor units and activity-dependent adaptations in type II muscle fibers—a speculation that requires direct testing via electromyography or muscle biopsy (Bassett and Howley, 2000). No functional data from this study support this chain of events; the interpretation remains a hypothesis to be tested in future mechanistic studies. (ii) This study found a suggestive association between rs757511 and the changes in VCO_2_ after HIIT (the AA genotype individuals showed the greatest increase). However, this statistical observation does not reveal the mechanism. VCO_2_ not only reflects the CO_2_ production of tissues but also the efficiency of CO_2_ transport from peripheral tissues to pulmonary capillaries (Habedank et al., 1998; Xi et al., 2014). Carbonic anhydrase (CA) catalyzes the reversible hydration of CO_2_ to form carbonic acid, which is necessary for the overall CO_2_ clearance. Based on the literature, the Kv3.1 channel does not directly regulate the expression or activity of CA; however, there is a hypothesis suggesting that the enhanced neural drive mediated by Kv3.1-dependent neurons may indirectly affect CA function by regulating the pH homeostasis within muscle cell (Dodgson et al., 1980; Mayeux et al., 1996). (iii) Association between rs757511 and HR_max training response: Parasympathetic control of heart rate is mediated by vagal acetylcholine release onto M_2_ muscarinic receptors in the sinoatrial (SA) node, leading to GIRK channel activation, membrane hyperpolarization, and bradycardia (Tsentssevitsky et al., 2022). Kv3.1 channels support high-fidelity, high-frequency firing in vagal efferent neurons; thus, their functional integrity enhances inhibitory vagal tone to the SA node (Kawada et al., 2010). Notably, AG heterozygotes showed a mean increase in maximal HR (Δ = +2.55 bpm), whereas both homozygote groups (GG and AA) exhibited decreases. However, this observation should be interpreted with great caution. The AG group had a large standard deviation (± 2.58 bpm), indicating substantial individual variability, and the result may be driven by a few outliers or regression to the mean. Given the small subgroup size and the absence of a non-exercise control group, this finding is more plausibly explained by statistical noise or sampling instability than by a true biological effect. We therefore refrain from proposing a specific molecular mechanism for this pattern and recommend independent replication in larger cohorts (Ottschytsch et al., 2002; McKeown et al., 2008; Bocksteins et al., 2014). These observations suggest that the functional impact of rs757511 does not conform to a simple additive genetic model; instead, heterozygosity may confer a distinct, non-intermediate phenotype—highlighting the nuanced pathophysiological consequences of ion channel gene variation. (iv) Association between rs61882396 and VO_2_/kg (RE) training response: Given the preferential expression of Kv3.1 in fast-twitch motor neurons, this channel likely contributes to activity-dependent skeletal muscle fiber-type plasticity. The AA genotype appears to augment HIIT-induced conversion of type IIx to type IIa fibers—a transition associated with elevated oxidative enzyme activity, improved contractile efficiency, and reduced oxygen cost during submaximal running (Bruseghini et al., 2019; Deshmukh et al., 2021). (v) Association between rs12574348 and HR_submax (RE) training respons: A reduction in submaximal heart rate during running economy assessment reflects enhanced cardiovascular efficiency—specifically, improved stroke volume and reduced chronotropic demand at a given workload. HIIT has been shown to upregulate SERCA2a expression and enhance phospholamban phosphorylation in cardiomyocytes, thereby accelerating diastolic calcium reuptake and improving ventricular filling efficiency (Kubo et al., 2003). The CC genotype may potentiate these molecular adaptations, resulting in greater augmentation of stroke volume and a correspondingly lower heart rate at equivalent cardiac output.

Given the lack of direct experimental evidence for the direct regulation of the Kv3.1 channel protein function at these three loci, we queried the HaploReg v4.1 database to assess the linkage disequilibrium (LD) relationship between them and the known functional sites within or near the *KCNC1* gene. No significant LD was observed in the CHB (Chinese Beijing Han) reference population. This suggests that they may represent independent association signals. Since the association analysis in this study is based on population statistical inference and the current limited functional annotation information, these SNPs are more likely to serve as markers linked to uncharacterized functional variations rather than direct functional effect sites. It should be noted that neither the HaploReg nor the GTEx databases have reported these SNPs as KCNC1 eQTL in skeletal muscle or neural tissues. Therefore, the interpretation of the molecular mechanism of the findings in this study should be cautious, and these associations should be regarded as exploratory results that need to be verified by independent functional studies.

### Mechanistic speculation

The mechanistic interpretations offered above are speculative and derive entirely from the known biology of Kv3.1 channels as reported in the literature. This study did not collect any functional data—no gene expression analysis, no muscle biopsies, no fiber typing, no electrophysiological recordings, no neuromuscular junction assessments, and no direct measures of autonomic tone or cardiac contractility. Therefore, the proposed pathways (e.g., enhanced neural drive, type II fiber remodeling, AMPK-PGC-1α activation, vagal modulation) are not demonstrated by the present data; they are merely plausible hypotheses that could explain the statistical associations if they were true. Readers should treat all mechanistic statements as hypothesis-generating rather than evidence-based conclusions.

## Research limitations

This study did not set up a non-exercise control group, so it is impossible to completely rule out the influence of natural physiological fluctuations, seasonal effects, or regression to the mean on the changes in indicators before and after training. However, the main scientific question of this study is to identify genetic markers related to training sensitivity, rather than precisely quantifying the absolute training effect of HIIT. In the association analysis, we included baseline indicators, age, gender, etc. as covariates in the linear regression model. This strategy to some extent controlled the potential influence of the aforementioned confounding factors. Nevertheless, future studies still need to include randomly assigned control groups to verify the robustness of this finding.

The *post hoc* power analysis revealed that, under the Bonferroni-corrected significance threshold (α = 0.00167), the efficacy of this study in detecting SNP associations with moderate effect size (R² ≈ 5%) was less than 10%. Therefore, the reported P-values in the range of 0.014 to 0.048 from this study did not reach the corrected significance level and should be strictly regarded as exploratory findings. These results require validation in independent, larger sample size cohorts.

## Conclusion

This study examined the association between SNPs in the *KCNC1* gene and interindividual variability in aerobic capacity improvements following a 12-week HIIT intervention. Three SNPs—rs12574348, rs757511, and rs61882396—demonstrated suggestively significant associations with post-intervention changes in key physiological parameters, including VO_2_max/kg, VCO_2_, HR, VO_2_/k (RE), and HR_submax (RE). Notably, no significant associations were observed between these SNPs and baseline values of the same parameters, suggesting that the identified variants modulate training responsiveness rather than constitutive aerobic function. Specifically, individuals homozygous for the C allele at rs12574348 (CC genotype) exhibited greater improvements in VO_2_max/kg and reductions in HR_submax (RE); carriers of the A allele homozygous genotype (AA) at rs757511 showed increased VCO_2_, whereas those with the GG genotype displayed decreased HR_max; and individuals with the AA genotype at rs61882396 demonstrated reduced VO_2_/kg (RE). These genotype–phenotype relationships may be mechanistically linked to the role of the Kv3.1 potassium channel—encoded by *KCNC1*—in regulating neuromuscular conduction efficiency and the excitability of GABAergic inhibitory neurons. However, this proposed mechanism remains speculative and warrants validation through targeted molecular and functional studies. Collectively, these findings suggest that *KCNC1* polymorphisms may serve as candidate genetic markers for predicting individual responsiveness to HIIT in healthy young Han Chinese adults. Further studies are required to determine whether these associations generalize to other ethnicities, age groups, and fitness levels before they can inform personalized exercise prescription strategies in broader populations.

## Data Availability

The original contributions presented in the study are included in the article/**Supplementary Material**. Further inquiries can be directed to the corresponding author.
